# The DSM Directive Two Years On: Do Things Ever Get Easier?

**DOI:** 10.1007/s40319-021-01082-6

**Published:** 2021-06-16

**Authors:** Eleonora Rosati

**Affiliations:** Professor of Intellectual Property Law and Director of the Institute for Intellectual Property and Market Law (IFIM), Stockholm University, Stockholm, Sweden

In 2019, the European Parliament and the Council adopted Directive 2019/790 on copyright and related rights in the Digital Single Market^1^ (DSM Directive), an ambitious piece of legislation supported by multiple rationales, including to: (1) guarantee a high level of protection; (2) streamline rights clearance; (3) create a level playing field for the exploitation of protected content; (4) remedy interpretative uncertainties; and (5) guarantee a well-functioning and fair marketplace for protected content. 7 June 2021 was – or rather, would have been – the deadline for the national transpositions of the DSM Directive.

Two things are certain in all this. The first is that most EU Member States failed to meet the June deadline. The Netherlands was the first country to transpose the Directive in full.^2^ Other Member States are also likely to finalise the transposition process before the summer recess period begins or in the course of Autumn 2021. However, in a significant number of countries, the process is still at relatively an early stage.^3^ The second thing is that, compared to when the Directive entered into force (7 June 2019), today’s Europe is a very different place, and so is the rest of the world. In 2019, Brexit was on the horizon, but no one knew what “Brexit means Brexit”^4^ actually meant. Now we know; we also know that the United Kingdom did not transpose this piece of EU legislation. In 2019, the abrupt and unprecedented global disruption that COVID-19 would cause only a few months later was also unforeseen and likely unforeseeable. The pandemic might have been *in part* responsible for delaying a number of events specifically surrounding the transposition process, with the result of stalling it. Such events *inter alia* include the release of the Commission’s guidance on the application of Art. 17, one of the most complex and discussed provisions in the DSM Directive.

Between 15 October 2019 and 10 February 2020, the Commission organised and held six stakeholder dialogue meetings to receive views regarding the application of Art. 17, in particular the cooperation referred to in paragraph 4 therein. Following these meetings, in summer 2020 the Commission launched a public consultation based on a document that, whilst building on the discussions held, also presented the initial views of the Commission itself.^5^ The plan was to finalise the Commission’s guidance in the second-half of 2020. The guidance was finally published on 4 June 2021.^6^ The Commission’s guidance is “soft law” and shall not be binding, as such, on the Court of Justice of the European Union (CJEU) when interpreting Art. 17.^7^ Insofar as national authorities and courts are concerned, the Commission’s guidance shall be taken into *consideration* in order to decide disputes submitted to them, because of the indirect effect that non-binding law has. In any event, in accordance with settled case law, the Commission’s Art. 17 guidance will not confer rights on individuals, which the latter may rely upon before such courts and authorities.^8^ This, in turn, means that the Commission guidance will not be enforceable per se before national courts. This does not rule out that the Commission’s guidance itself might be the subject of referrals for a preliminary ruling: since the judgment in *Grimaldi* (C-322/18) the CJEU “has consistently insisted that Article 267 TFEU confers on the Court jurisdiction to give a preliminary ruling on both the validity and the interpretation of all acts of the institutions of the Union ‘without exception’”.^9^

Besides the delayed Art. 17 guidance, further events which are due to have an impact on the DSM Directive and its national (transpositions and) applications are also two forthcoming CJEU decisions. The first is the (at the time of writing, pending) ruling in joined cases *YouTube* (C-682/18) and *Cyando* (C-683/18): albeit that these referrals were not, nor could have been, on the DSM Directive, the relationship between Art. 3 of the InfoSoc Directive^10^ and Art. 17 of the DSM Directive was discussed during the hearing and held a prominent role in the analysis contained in the 2020 Opinion of Advocate General (AG) Saugmandsgaard Øe. With regard to the right of communication/making available to the public, unlike the Commission in its finalised Art. 17 guidance, the AG held the view that, whilst the platforms at issue in the background proceedings (YouTube and cyberlocker Uploaded) would be liable in principle under Art. 17 lacking a licence, the same would not be true under Art. 3 alone.^11^ In this sense, Art. 17 of the DSM Directive would be a novel regime, which does not have retroactive application.^12^ Turning to the safe harbour availability, the AG advised the CJEU to rule that the privilege in Art. 14 of the Ecommerce Directive^13^ – at least prior to the adoption of the DSM Directive (see Art. 17(3)) – would in principle be available to platforms like those at issue in the background proceedings, irrespective of the type of liability at hand.^14^ The second judgment is the one to be rendered in the Polish challenge to Art. 17 of the DSM Directive.^15^ The action of the Republic of Poland is based on an alleged breach of the right to freedom of expression and information as *inter alia* guaranteed by Art. 11 of the EU Charter of Fundamental Rights.^16^ The AG Opinion in this case, originally due for release on 22 April 2021, is now expected to be published on 15 July 2021. If this schedule is maintained, then the CJEU judgment regarding the validity of one of the most relevant provisions of the Directive will be likely rendered in early 2022.

In all this, also the discussion surrounding further proposed EU legislation will have, without doubt, an influence on the application of the DSM Directive, notably – once again – its Art. 17. In late 2020, the Commission unveiled its Proposal for a Digital Services Act (DSA).^17^ Whilst being expressly without prejudice to Union law in the field of copyright and related rights, including the DSM Directive, it is evident that the finalisation of the DSA will be also relevant to the interpretation and application of Art. 17 of the DSM Directive, including with regard to safe harbour availability and the prohibition of general monitoring, as well as trusted flaggers, transparency and notice requirements.

In sum, and to conclude: the story of the DSM Directive *before* its adoption is a complex one. Some of its provisions were the subject of heated debates, and their resulting wording owes to political and legal compromise. This, in turn, means that they are also complex provisions and that the story of the DSM Directive *after* its adoption will by no means be a straightforward one. The events described above are probably just a small glimpse into the future: litigation concerning the national provisions by which Member States have transposed or will transpose the DSM Directive is around the corner, and so are CJEU referrals from national courts. This is not a bad thing, nor is it something that casts shadows on the merits and quality of the EU copyright law-making process. Rather, it is testimony to the complexity of copyright, the variety of (contrasting) interests underlying policy- and law-making in this area, and the growing realisation that the incremental dialogue between legislature and courts – on both the EU and national level – is both unavoidable and welcome. So, no, things will not get any easier. Hopefully, however, they will become clearer ... in the end.

## Footnotes

1 Directive (EU) 2019/790 of the European Parliament and of the Council of 17 April 2019 on copyright and related rights in the Digital Single Market and amending Directives 96/9/EC and 2001/29/EC, OJ L 130, 17.5.2019, pp. 92–125.

2 Staatsblad van het Koninkrijk der Nederlanden, stb-2020-558, 29 December 2020.

3 A useful tracker is the one developed by Communia: *see* https://www.notion.so/DSM-Directive-Implementation-Tracker-361cfae48e814440b353b32692bba879 (last accessed 4 June 2021).

4 This phrase was pronounced by former UK Prime Minister Theresa May in 2016: *see* https://www.bbc.com/news/av/uk-politics-36764525 (last accessed 4 June 2021).

5 Targeted consultation addressed to the participants to the stakeholder dialogue on Art. 17 of the Directive on Copyright in the Digital Single Market (2020), available at https://ec.europa.eu/digital-single-market/en/news/directive-copyright-digital-single-market-commission-seeks-views-participants-stakeholder (last accessed 4 June 2021).

6 European Commission, *Communication from the Commission to the European Parliament and the Council, Guidance on Article 17 of Directive 2019/790 on Copyright in the Digital Single Market*, Brussels, 04.06.2021, COM(2021) 288 final.

7 Article 267 of the Consolidated version of the Treaty on the Functioning of the European Union, OJ C 326, 26.10.2012, pp. 47–390 (TFEU).

8 *Grimaldi*, C-322/88, EU:C:1989:646, at [19].

9 Opinion of Advocate General Bobek in *FBF*, C-911/19, EU:C:2021:294, at [32].

10 Directive 2001/29/EC of the European Parliament and of the Council of 22 May 2001 on the harmonisation of certain aspects of copyright and related rights in the information society, OJ L 167, 22.6.2001, pp. 10–19.

11 *Contra* and opining that the CJEU would not follow the AG Opinion, *see* also *Capitol Records & Ors v. British Telecommunications Plc & Ors* [2021] EWHC 409 (Ch) (25 February 2021), at [26]–[27]; and *Young Turks Recordings Ltd & Ors v. British Telecommunications Plc & Ors* [2021] EWHC 410 (Ch) (25 February 2021), at [80].

12 Opinion of Advocate General Saugmandsgaard Øe in *YouTube*, C-682/18 and *Cyando*, C-683/18, EU:C:2020:586, at [66]–[93].

13 Directive 2000/31/EC of the European Parliament and of the Council of 8 June 2000 on certain legal aspects of information society services, in particular electronic commerce, in the Internal Market, OJ L 178, 17.7.2000, pp. 1–16.

14 Opinion of Advocate General Saugmandsgaard Øe in *YouTube*, C-682/18 and *Cyando* C-683/18, EU:C:2020:586, at [138].

15 *Poland v. Parliament and Council*, C-401/19 (in progress).

16 Charter of Fundamental Rights of the European Union, OJ C 326, 26.10.2012, pp. 391–407.

17 Proposal for a Regulation of the European Parliament and of the Council on a Single Market For Digital Services (Digital Services Act) and amending Directive 2000/31/EC, COM/2020/825 final.

